# Supplementary material for: Modulation of the Drosophila transcriptome by developmental exposure to alcohol
Source: BMC Genomics. 2022 May 6;23:347. doi: 10.1186/s12864-022-08559-9 (PMC9074282; doi:10.1186/s12864-022-08559-9)
Supplement: Supplementary file 6 — Additional file 6. snoRNAs with altered gene expression following chronic exposure to ethanol during development and their host genes. snoRNAs that occur in clusters without intervening genes are in bold font and square brackets. [file 12864_2022_8559_MOESM6_ESM.docx]

**Additional File 6: snoRNAs with altered gene expression following chronic exposure to ethanol during development and their host genes.** snoRNAs that occur in clusters without intervening genes are in bold font and square brackets.

**Host Gene Function Number of snoRNAs snoRNAs Responsive to Chronic Ethanol Exposure**

*dom*  histone acetyltransferase activity 16 snoRNAs in multiple introns *snoRNA:Psi18S-841d - snoRNA:Psi18S-1389a*

and 1 snmRNA

*RpL5*  ribosomal protein L5 1 snoRNA in intron *snoRNA:Psi28S-2996*

*RpS5a* ribosomal protein S5a 14 snoRNAs in multiple introns **[*snoRNA: Psi28S-1135a - snoRNA: Psi28S-1135b -snoRNA:***

***Psi28S-1135c - snoRNA: Psi28S-1135d -snoRNA*** ***Psi18S-1854a*]** *snoRNA:Psi28S-1854c - snoRNA: Psi28S-1192a*

*Uhg4*  non protein coding gene, ncRNA 7 snoRNAs *snoRNA:OR-CD2*

*CG13900*  (*Sf3b3*; Splicing factor 3b subunit 3) 9 snoRNAs in multiple introns snoRNA: Psi18S-1086 - *snoRNA: Psi28S-3308 - snoRNA18S-110*

*RpL17* ribosomal protein L17 4 snoRNAs in multiple introns *snoRNA: Psi28S-1060*

Between *GlyT*  1 snoRNA *snoRNA: Psi18S-176*

and *CR44805*

*kra* translation initiation factor binding 8 snoRNAs in multiple introns *snoRNA: Psi28S-3186 - snoRNA: Psi28S-3091a -* *snoRNA: Psi28S-3385a*

*RpL22/CR42491* ribosomal protein 22 (CR42491 nested) 2 snoRNAs in the intron *snoRNA: Psi18S-531*

*Aladin* nuclear pore complex 1 snoRNA in intron *snoRNA: CG16892-a*

*Msp300* Muscle-specific protein 300 kDa 2 snoRNAs and 3 snmRNAs *snoRNA: Psi18S-525k*

*Nop60B*  pseudouridine synthase activity 7 snoRNAs in multiple introns *snoRNA: Psi18S-1820*

*stet*  serine type endopeptidase 1 snoRNA and 1 snmRNA in intron *snoRNA: Psi28S-2622*

*RpS7* ribosomal protein S7 7 snoRNAs in the same intron **[*snoRNA: Psi18S-1377c - snoRNA: Psi18S-1377d - snoRNA:***

***Psi18S-1377e*]**

*Sc35* SR family splicing factor SC35 2 snoRNAs in the same intron *snoRNA: SC35-a*

*CG1646* pre-mRNA 5'-splice site binding 1 snoRNA in an intron *snoRNA: 291*

*lds* helicase, maintains chromosome stability 1 snoRNA in an intron *scaRNA: MeU2-C41*

*CG10576* Winged helix-turn-helix DNA-binding domain 5 snoRNAs in multiple introns *snoRNA: Psi28S-1837c*

and 1 snmRNA

*RpS4*  ribosomal protein S4 3 snoRNAs in multiple introns *snoRNA: Psi28S-3327b*

*pAbp* poly(A) binding protein 3 snoRNAs in the same intron *snoRNA: Psi18S-1347a*

*RpS16* ribosomal protein S16 5 snoRNAs in multiple introns **[*snoRNA: Psi28S-1175a - snoRNA: Psi28S-1175b -***

***snoRNA: Psi28S-1175c - snoRNA: Or-aca1*]**

*RpL11*  ribosomal protein L11 1 snoRNA in an intron *snoRNA:Psi28S-2444*

*Pi4KIIalpha* Pi4KIIα synthesizes 1 snoRNA in an intron *snoRNA: Pi4KIIalpha-a*

phosphatidylinositol 4-phosphate
