# Supplementary figures and images for: Modulation of the Drosophila transcriptome by developmental exposure to alcohol
Source: BMC Genomics. 2022 May 6;23:347. doi: 10.1186/s12864-022-08559-9 (PMC9074282; doi:10.1186/s12864-022-08559-9)

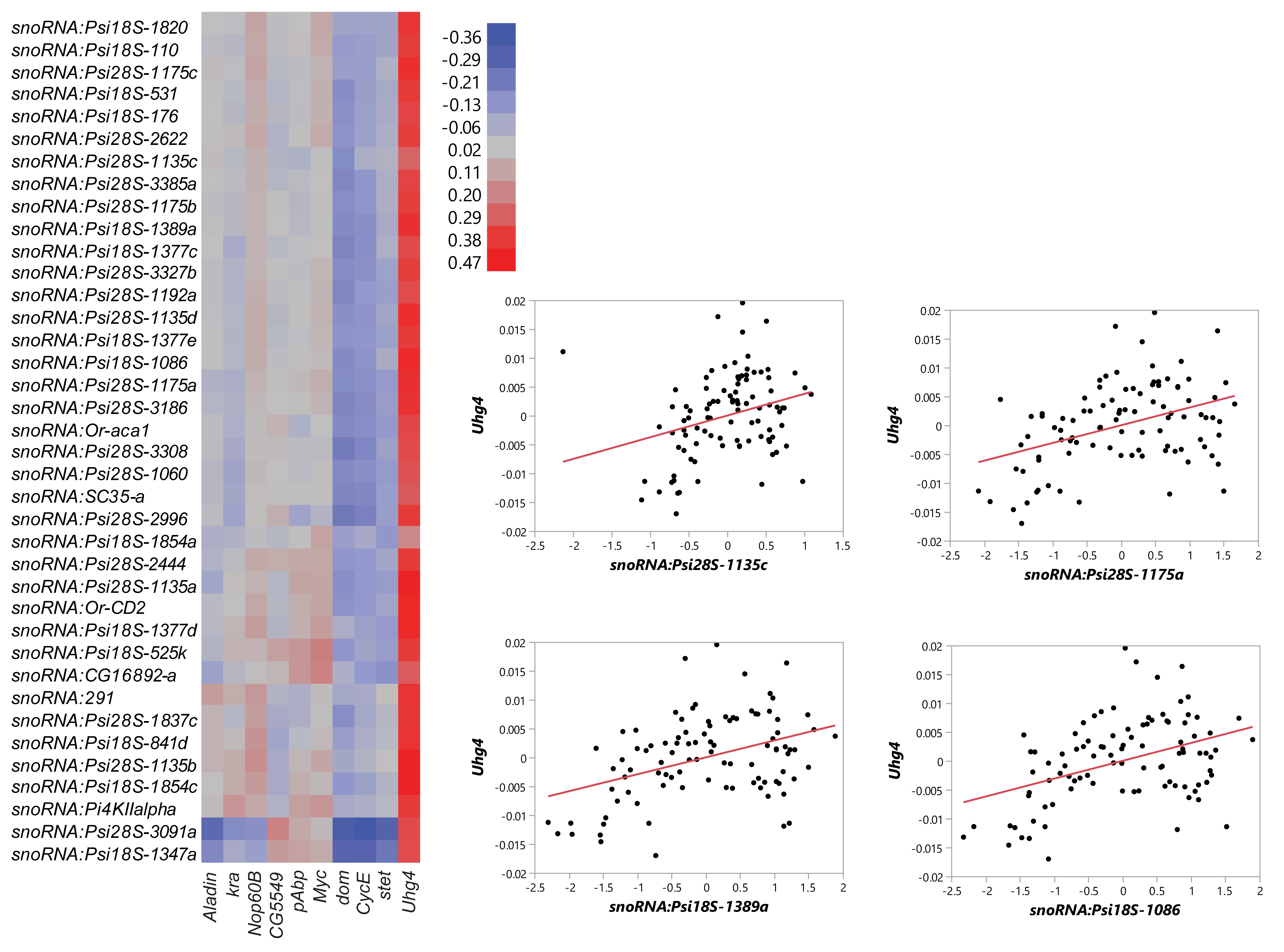

Supplement: Supplementary file 8 — Additional file 8: Figure S1. Correlations between variation in expression of snoRNAs after chronic exposure to ethanol and variation in expression of their host genes. CycE is included since it is a hub gene in a genetic network associated with variation in ethanol-induced variation in development time and viability [16], and Myc is included as it is associated with ribosome biogenesis and has been implicated as a regulator of Uhg4 [38]. The graphs on the right illustrate examples of scatter plots of the correlations between expression of Uhg4 and several snoRNAs. [file 12864_2022_8559_MOESM8_ESM.tif]

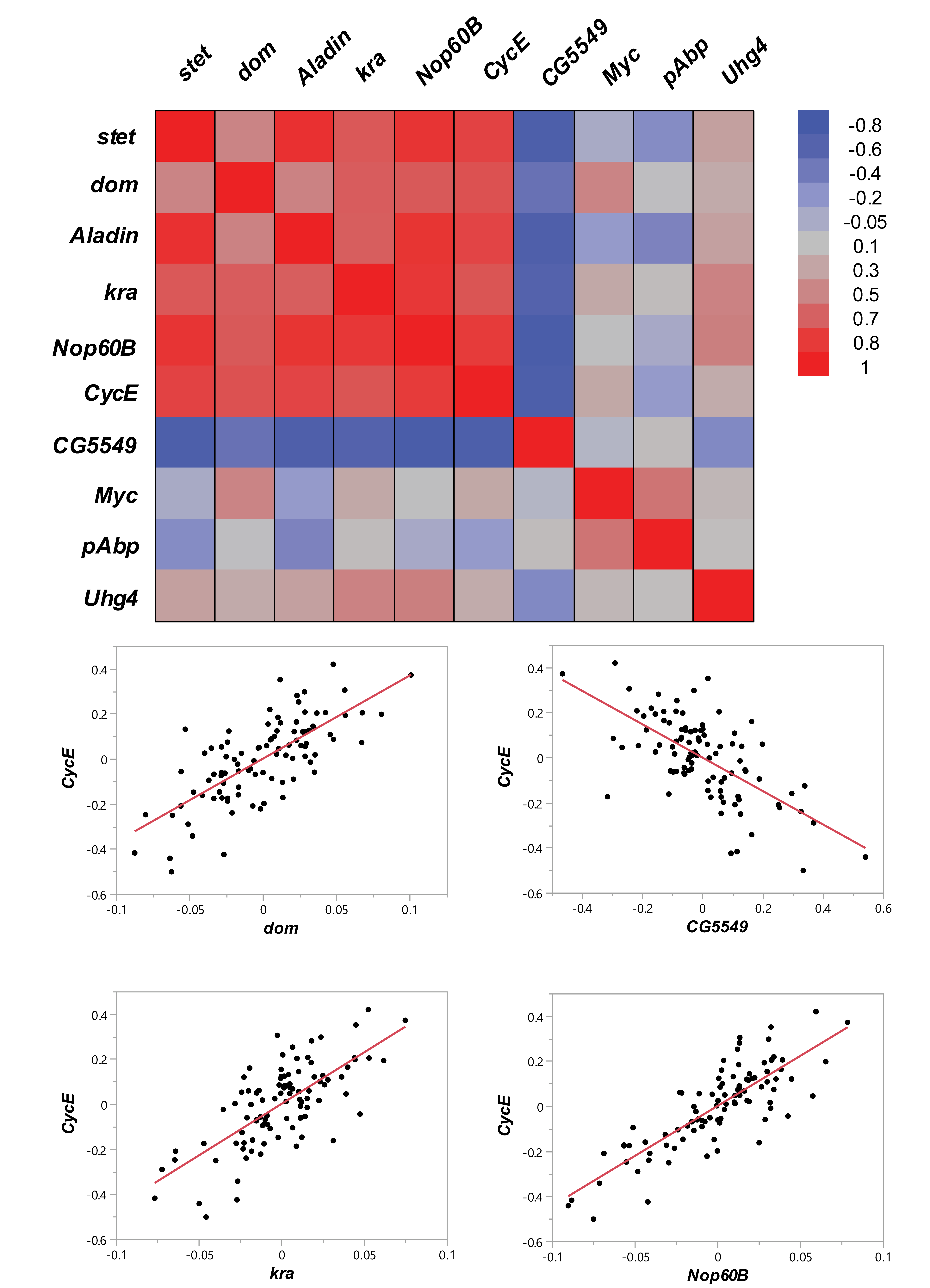

Supplement: Supplementary file 9 — Additional file 9: Figure S2. Correlations between ethanol-induced variation in expression of snoRNA host genes and variation in expression of CycE. The scatter plots illustrate examples of correlations between expression of CycE and several snoRNA host genes. [file 12864_2022_8559_MOESM9_ESM.tif]
